# Policy disparities in response to COVID-19 between Singapore and China

**DOI:** 10.1186/s12939-021-01525-z

**Published:** 2021-08-17

**Authors:** Xiaohan Wang, Leiyu Shi, Yuyao Zhang, Haiqian Chen, Gang Sun

**Affiliations:** 1 Department of Health Management, School of Health Management, Southern Medical University, Guangzhou, Guangdong 510515, PR China; 2 Department of Health Policy and Management, Bloomberg School of Public Health, Johns Hopkins University, Baltimore, MD 21205 USA

**Keywords:** Global health equity, COVID-19, Control policies, Isolation, Case detection

## Abstract

**Objective:**

The study analyzed the common points and discrepancies of COVID-19 control measures of the two countries in order to provide appropriate coping experiences for countries all over the world.

**Method:**

This study examined the associations between the epidemic prevention and control policies adopted in the first 70 days after the outbreak and the number of confirmed cases in China and Singapore using the generalized linear model. Policy comparisons and disparities between the two countries were also discussed.

**Results:**

The regression models show that factors influencing the cumulative number of confirmed cases in China: Locking down epicenter; activating Level One public health emergency response in all localities; the central government set up a leading group; classified management of “four categories of personnel”; launching makeshift hospitals; digital management for a matrix of urban communities; counterpart assistance. The following four factors were the key influencing factors of the cumulative confirmed cases in Singapore: The National Centre for Infectious Diseases screening center opens; border control measures; surveillance measures; Public Health Preparedness Clinics launched.

**Conclusions:**

Through analyzing the key epidemic prevention and control policies of the two countries, we found that the following factors are critical to combat COVID-19: active case detection, early detection of patients, timely isolation, and treatment, and increasing of medical capabilities. Countries should choose appropriate response strategies with health equity in mind to ultimately control effectively the spread of COVID-19 worldwide.

## Introduction

The novel coronavirus disease 2019 (COVID-19) was first reported in Wuhan, China, on 31 December 2019, and has rapidly spread throughout China in just several months [1]. China is the most populous country in the world, with over 1.4 billion people living in a land area of about 9.6 million square kilometers (Sq. Km). The population density of China is 147.8 People Per Sq. Km and the average household size is 3.10 persons [2, 3]. The Chinese government promptly established a rapid and effective epidemic emergency mechanism and adopted a series of prevention and control policies to significantly reduce the spread of the epidemic. Since mid-February, the daily number of new COVID-19 cases has been declining in China [4]. The World Health Organization (WHO) confirmed that the epicenter of the outbreak was originally in China and had been transferred to European countries on 13 March 2020, and then that label had shifted to New York [5].

Singapore is a densely populated city-state of 5.7 million, and as a major air hub had an average of 330,000 visitor arrivals from China each month in 2019 [6]. According to the data released by the World Bank in 2018, Singapore has a land area of 709 Sq. Km and a high population density of 7952.9 People Per Sq. Km. The average household size is 3.16 persons which are similar to China [2, 7]. These characteristics make Singapore one of the countries hit earlier by the epidemic. The first COVID-19 confirmed case was reported in Singapore as early as 23 January 2020. In 2003, the severe acute respiratory syndrome (SARS) outbreak in Guangdong, China, and Singapore was also one of the most heavily affected areas. Learning from the SARS outbreak, the Chinese government formulated and implemented the “Regulations on Preparedness for the Response to Emergent Public Health Hazards”, and Singapore strengthened its ability to manage emerging infectious disease outbreaks including developing a national pandemic preparedness plan [8]. To reduce the spread of COVID-19, Singapore has rapidly adopted containment measures. Until the end of March 2020, the number of new cases in Singapore remained at a low level, and there was only one death.

China and Singapore, as early-hit nations, have gained international recognition for their effectiveness in epidemic prevention and control. We systematically summarized epidemic control measures of China and Singapore and carried out a quantitative analysis to investigate associations between the epidemic control measures and the number of COVID-19 cases reported in China and Singapore outbreaks in the first 70 days. The study analyzed the common points and discrepancies of COVID-19 control measures of the two countries in order to provide appropriate coping experiences for countries all over the world.

## Methods

### Data collection

Data on COVID-19 cases in China were obtained from the National Health Commission of the People’s Republic of China, which provides daily updates with case data on web-page. Singapore’s epidemic data were available in the situation report of the Ministry of Health Singapore. The National Health Commission of the People’s Republic of China and the Ministry of Health Singapore are both authoritative official government websites with a certain guarantee of data quality. We compiled and integrated data for the first 70 days after the COVID-19 outbreak in China and Singapore, respectively. The research indicators included the cumulative number of laboratory-confirmed cases, the number of new cases, and the number of new deaths.

### Statistical analysis

We examined the associations between the epidemic prevention and control policies adopted in the first 70 days after the outbreak and the number of confirmed cases in China and Singapore using the generalized linear model, respectively. The cumulative number of confirmed cases was taken as dependent variables Y, and the policies summarized in Tables 1 and 2 were respectively entered into generalized linear models as independent variables X. China and Singapore were regarded as implementing an intervention when the official policy was announced publicly. All data-sorting was conducted using Microsoft Excel 2019, and statistical data was analyzed by IBM SPSS.

**Table 1:** Seven major prevention and control policies in China

SN	Date	Policy	Key elements
1	23-Jan	Locking down epicenter	The government put the Wuhan under lockdown by banning travel to and from the city, as well as suspending all public transport services in the city, and then locked down the whole Hubei Province.
2	24-Jan	Activating Level One public health emergency response in all localities	China entered a highly alert state of the national epidemic along with a series of public health response measures.
3	25-Jan	The central government set up a leading group	The Central Government established a Leading Group on coping with the COVID-19 and sent guidance teams to Hubei and other hard-hit areas.
4	2-Feb	Classified management of “four categories of personnel”	Wuhan put four categories of people – confirmed cases, suspected cases, febrile patients who might be carriers, and close contacts – under classified management in designated facilities. The measure was subsequently carried out nationwide.
5	5-Feb	Launching makeshift hospitals	Launching Leishen Shan Hospital and Huoshen Shan Hospital for patients in severe or critical condition. Between Feb 5 and Mar 10, Wuhan had opened a total of 16 Fangcang shelter hospitals for patients with mild symptoms.
6	10-Feb	Digital management for a matrix of urban communities	Residential communities remained under closed-off management in Wuhan. Digital management for a matrix of urban communities across the China.
7	12-Feb	Counterpart assistance	Through “One Province Supports One City”, the entire country will support the epidemic prevention and control in Hubei Province.

**Table 2:** seven major prevention and control policies in Singapore

SN	Date	Policy	Key elements
1	23-Jan	Setting up a Multi-Ministry Task Force	The preliminary plan of the Multi-Ministry Task Force, drawn up after the 2003 SARS outbreak, was launched on 23 January 2020 to coordinate among departments and provide strategic and political guidance during the public health crisis.
2	29-Jan	NCID screening center opened	The National Centre for Infectious Diseases (NCID), a 330-bed purpose built infectious disease management facility with integrated clinical, laboratory and epidemiological functions, enhanced Singapore’s infrastructure for outbreak management.
3	1-Feb	Border control measures	Singapore was stepping up border control measures to prevent importation of cases and forward local transmission, from temperature and health screening of incoming travellers to entry restrictions of travellers in countries with epidemic outbreak.
4	4-Feb	Social and community measures	The social and community measures was focused on social responsibility while life continued as usual with precautions, including public education, the use of masks, monitoring employees’ temperature and health regularly and so on.
5	5-Feb	Expanding SARS-CoV-2 testingcapacities	RT-PCR laboratory testing capability was expanded from the National Public Health Laboratory to all public hospitals in Singapore, allowing more than 8000 tests to be performed daily.
6	7-Feb	Surveillance measures	Every COVID-19 case and close contact was tracked as closely as possible through several complementary detection methods.
7	14-Feb	PHPCs launched	A network of > 800 Public Health Preparedness Clinics (PHPCs) that provide subsidized care and extended medical leave of up to 5 days was activated to enhance management of respiratory infections in the primary care setting.

## Results

### Major prevention and control policies in China and Singapore

**Fig. 1:**
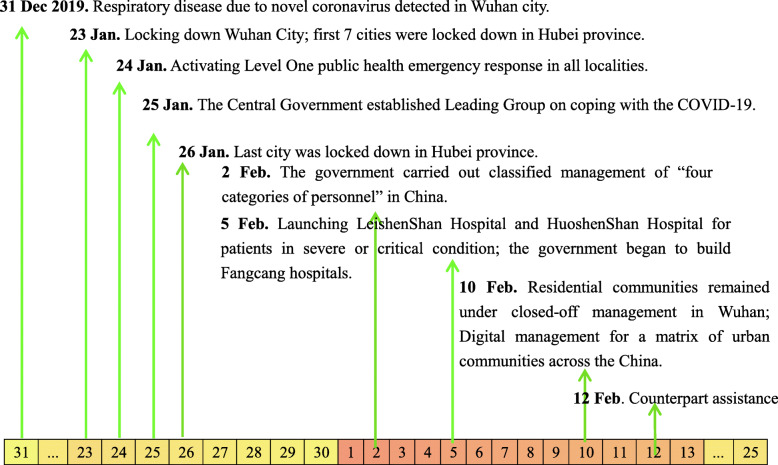
Dates of discovery of the novel coronavirus and the implementation of control measures in China, from 23 January 2020

The Chinese government has taken serious nationwide response measures which were shown in Fig. 1 to fight a COVID-19 outbreak, and we summarized them in the following major seven items (Table 1): locking down epicenter; activating Level One public health emergency response in all localities; the central government set up a leading group; classified management of “four categories of personnel”; launching makeshift hospitals; digital management for a matrix of urban communities; counterpart assistance.

**Fig. 2:**
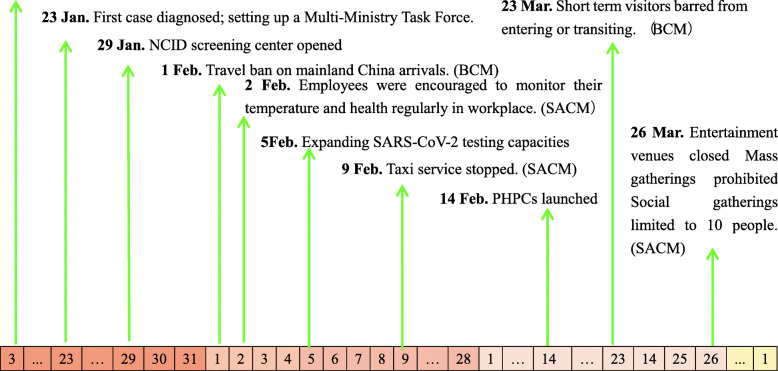
the implementation of control measures in Singapore, from3 January 2020

Singapore’s approaches to reducing the transmission of COVID-19 were shown in Fig. 2 and summarized in Table 2: setting up a Multi-Ministry Task Force; The NCID screening center opened; border control measures; social and community measures; expanding SARS-CoV-2 testing capacities; surveillance measures; PHPCs launched.

### Epidemiological timeline for the first 70 days of COVID-19 in China and Singapore

**Fig. 3:**
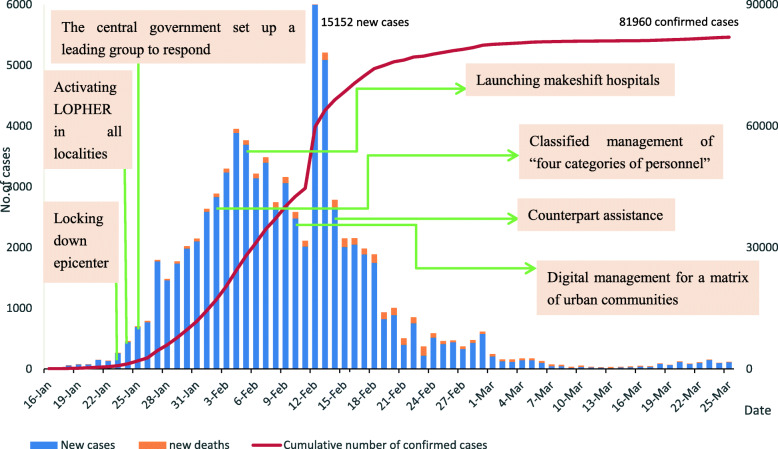
Epidemiological timeline of COVID-19 in China, and major transmission control measures. Note: Epidemiological timeline of COVID-19 in China from January 16 to March 25, and major transmission Control measures are marked only with the start date. LOPHER: Level One public health emergency response. Adapted from National Health Commission of the People’s Republic of China. Situation report -update on COVID-19 as of 25 March 2020. http://www.nhc.gov.cn/xcs/yqtb/202003/f01fc26a8a7b48debe194bd1277fdba3.shtml

Data on laboratory-confirmed cases began to be daily announced on the official website of the National Health Commission of the People’s Republic of China from 16 January 2020. Figure 3 shows the epidemiological timeline for the first 70 days of COVID-19 in China and major prevention and control policies. With the major control measures, the newly confirmed cases showed a downward trend after the rapid increase from 16 January 2020 to 12 February 2020. The number of newly confirmed cases has been effectively controlled as the continuous maintenance of about 100 new cases after March 2020. The number of new deaths fluctuated slightly but remained at a low level.

**Fig. 4:**
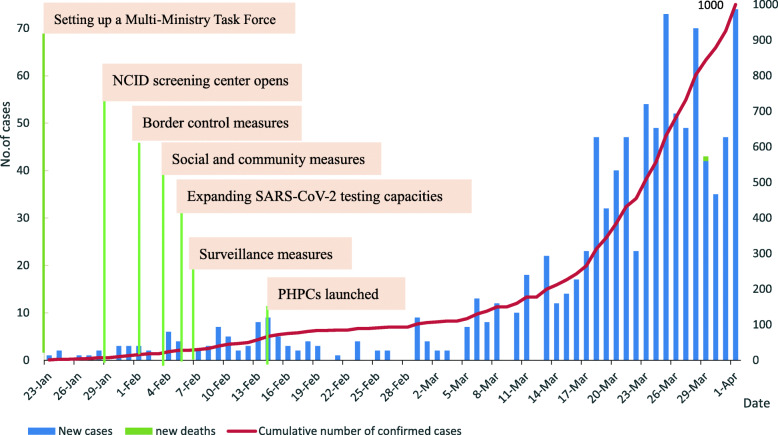
Epidemiological timeline of COVID-19 in Singapore and major transmission control measures. Note: Epidemiological timeline of COVID-19 in Singapore from January 23 to April 1, and major transmission Control measures are marked only with the start date. PHPC = Public Health Preparedness Clinic; NCID = National Centre for Infectious Diseases. Adapted from Ministry of Health Singapore. Situation report – 1 April 2020: coronavirus disease (COVID-19). https://www.moh.gov.sg/news-highlights/details/five-more-cases-discharged-74-new-cases-of-covid-19-infection-confirmed

Figure 4 shows the epidemiological timeline for the first 70 days of COVID-19 in Singapore and major control measures. Within 70 days after the confirmed cases appeared, relative normalcy of day-to-day life had been maintained in Singapore, and the government did not implement school closures or other major social-distancing measures. As of 1 April 2020, Singapore had only one death case. The number of newly confirmed cases was gradually increasing, but the overall trend remained relatively stable.

### Associations between the major prevention and control policies and the number of confirmed cases

The cumulative confirmed case in China is used as the dependent variable Y of the generalized linear model, and the seven major control measures 1–7 in Table 1 are used as the independent variables X1-X7 of the generalized linear model. Table 3 shows the results of the generalized linear model analysis, indicating that the seven main epidemic prevention and control measures taken in China have an impact on the number of confirmed cases. The cumulative confirmed case in Singapore is used as the dependent variable Y of the generalized linear model, and the seven major control measures 1–7 in Table 2 are used as the independent variables X1-X7 of the generalized linear model. Table 4 suggests that the following four factors were the key influencing factor of the cumulative confirmed cases: NCID screening center opens; border control measures; Surveillance measures; PHPCs launched.

**Table 3:** Associations between the major prevention and control policies and the number of COVID-19 cases reported in China outbreaks the first 70 days

Parameter	B	95%CI	Wald Chi-Square	Sig.
(Intercept)	11.27	(11.26, 11.27)	426,007,383.9	0.000
Locking down epicenter	−1.21	(−1.29, −1.13)	824.457	0.000
Activating Level One public health emergency response in all localities	−0.44	(−0.53, −0.35)	97.087	0.000
The central government set up a leading group	−1.74	(− 1.79,-1.69)	3822.159	0.000
Classified management of “four categories of personnel”	−1.03	(−1.05, − 1.02)	32,227.198	0.000
Launching makeshift hospitals	−0.51	(− 0.51,-0.49)	11,591.493	0.000
Digital management for a matrix of urban communities	−0.24	(−0.25,-0.24)	3422.239	0.000
Counterpart assistance	−0.58	(−0.58,-0.57)	28,764.257	0.000

**Table 4:** Associations between the major prevention and control policies and the number of COVID-19 cases reported in Singapore outbreaks the first 70 days

Parameter	B	95%CI	Wald Chi-Square	Sig.
(Intercept)	5.649	(5.63, 5.67)	435,182.368	0.000
Setting up a Multi-Ministry Task Force	0^a^	*	*	*
NCID screening center opens	−0.959	(−1.50,-0.42)	11.969	0.001
Border control measures	−0.55	(−0.99,-0.10)	5.756	0.016
Social and community measures	−0.325	(−0.81,-0.16)	1.739	0.187
Expanding SARS-CoV-2 testing capacities	−0.154	(−0.63,-0.32)	0.399	0.527
Surveillance measures	−0.436	(−0.72,-0.15)	8.969	0.003
PHPCs launched	−1.881	(−1.99,-1.77)	1049.255	0.000

## Discussion

Figures 3 and 4 show that the increase in confirmed cases and deaths in both China and Singapore was under control in the first 70 days after the outbreak of the COVID-19 epidemic. Control measures have been proved in practice, greatly reducing the spread of the epidemic. From the analysis of the prevention and control measures adopted by the two countries, China has adopted stricter measures than Singapore and needs stronger government control. Wuhan city, as the epicenter of the epidemic, was first locked down by the government, and the lockdown radius was expanded to all other cities in the Hubei province, encompassing 45 million population [9]. All residents were restricted to stay at home in self-quarantine, in order to prevent the spread of the virus. However, it is worth noting that strict mobility restrictions can have an unequal impact on vulnerable groups and those in the lowest power strata of societies. In comparison, Singapore has not implemented school closures or other major social-distancing measures in the first 70 days. Residents maintained relative normalcy of day-to-day life in Singapore.

China has adopted more radical and strict closed management measures, and Singapore has adopted more moderate precautions on the basis of maintaining a relatively normal social life. Although it seems that the measures taken by the two countries are not similar, but combined with the analysis results of the generalized linear model, we can see that the prevention and control policies of the two countries have a lot in common.

### Increasing medical capabilities

This study found that during the first 70 days of COVID-19 outbreak, NCID screening center and PHPCs had a significant impact on the cumulative confirmed cases in Singapore. One of the lessons learned from SARS is building the National Centre for Infectious Diseases (NCID). In order to strengthen Singapore’s capacity in infectious disease management and prevention, the 330-bed NCID is closely linked to the Chen Dusheng Hospital, which provides some medical staff. This enables NCID to accommodate nearly 500 beds in the outbreak, achieving inter-agency collaboration and coordinated operation [10]. On 29 January 2020, NCID opened the screening center to screen suspected cases. If high-risk patients were found, they were immediately quarantined. Low-risk suspected cases were monitored by telephone during home isolation. Public Health Preparedness Clinics (PHPCs), a network of over 800 primary health clinics that manage respiratory infections. At the same time, the government provided subsidies to Singapore residents to encourage them to seek care at these PHPCs. Central broadcasting network [11] Medical practitioners can provide 5 days of medical leave for patients with respiratory disorders, which allows patients with mild COVID-19 to isolate at home and reduce the risk of community transmission. If the patient’s symptoms worsen, the referral is made through PHPCs.

We also found that makeshift hospitals and counterpart assistance had a significant impact on the cumulative confirmed cases in China. Wuhan, as the epicenter of China, experienced a sharp increase in cases at the outbreak. Wuhan rushed to build Huoshenshan and Leishenshan temporary hospitals to treat severe patients and solve the problem of insufficient medical resources. The two hospitals are similar to Beijing’s Xiaotangshan temporary hospital, which fought against SARS in 2003, and was used to treat patients with severe diseases. Between Feb 5 and Mar 10, 2020, Wuhan had opened a total of 16 Fangcang shelter hospitals for patients with mild symptoms. The 16 Fangcang shelter hospitals admitted up to 12,000 patients with mild symptoms, accounting for more than a quarter of the infected patients [12]. Hubei, as a province whose economic and medical resources are highly biased towards Wuhan, was also facing a severe situation at the outbreak. There was a shortage of medical staff and hospitals were overloaded. The National Health Committee announced the establishment of a “one-to-one support relationship” among 16 provinces to support cities outside Wuhan. It is worth mentioning that through “One Province Supports One City”, China ensured the availability of medical resources in Hubei Province [13]. The two measures, makeshift hospitals, and counterpart assistance, complement each other to ensure China’s medical capabilities in this epidemic.

Whether Singapore launches NCID and PHPCs, or China builds Huoshenshan Hospital, Leishenshan Hospital, Fangcang shelter hospital and starts the counterpart support mode, all of them are in essence to enhance the medical capabilities to avoid a run on medical resources. Through sound primary health care settings and screening measures for suspected cases, Singapore can detect confirmed patients as early as possible and arrange for isolation or admission according to different conditions. Health expenditure per capita in Singapore in 2018 was USD$2823.638, compared to USD$501.059 in China [14]. As a country with a large population, China’s per capita health resources are indeed inadequate. Wuhan also had a large backlog of patients during the outbreak. It is difficult to ensure adequate medical resources in a city with such a serious outbreak. To speed up the clearance of the backlog and alleviate the further spread of the epidemic, the Chinese government resolutely mobilized national resources to build several makeshift hospitals for patients. Moreover, all the provinces in China were trying their best to assist other prefecture-level cities in Hubei, so as to improve the local medical capabilities and alleviate the shortage of medical resources in these cities.

Furthermore, all confirmed patients in Wuhan, regardless of whether they come from urban or rural residences, were covered by the health insurance fund and government subsidies at an average rate of 65% [15]. Low-income and disadvantaged populations are vulnerable to greater harm in infectious disease outbreaks. Government funding of medical treatment for COVID-19 patients ensures maximum health equity for the country’s population. The policy also applied to confirmed patients in other areas of China, and this initiative had guaranteed health equity within China. Countries around the world should increase medical capabilities to deal with the surge in patients. Please also note that as the “Buckets Effect” reveals: the capacity of a bucket depends on the shortest wooden board [16]. It is important to have sufficient health care resources, and it is also important to rationalize the deployment of health resources to ensure health equity in the country.

### Active case detection

The study found that border control measures and surveillance measures were effective in Singapore. The earliest border control measures were temperature and health screening of travelers from Wuhan to prevent imported cases and extended to all travelers since 29 January 2020 [17]. Travelers who meet the suspect case definition are conveyed directly to hospital. From 23 March, all short-term visitors were banned from entering Singapore, and residents were advised to postpone all non-essential travel [18]. Singapore’s surveillance for COVID-19 aimed to identify every case using multi-pronged strategies. The case definition was established by the government to identify suspect cases at healthcare facilities or through contact tracing. Patients who meet the criteria are referred by primary medical clinics to NCID or hospital emergency departments. Clinics and hospitals across the country conduct screening according to the government-issued criteria for suspected cases of pneumonia in COVID-19. Patients who meet the criteria are referred by primary medical clinics to NCID or hospital emergency departments. The Singapore government has also developed a mobile app “trace together” to help close contact track [19]. Furthermore, Singapore enhanced surveillance system among different patient groups (all cases of pneumonia in hospital and primary care, severely ill patients in hospital ICUs and deaths with possible infectious causes, and influenza-like illness in sentinel primary care clinics) [20].

The study also found that the classified management of “four categories of personnel” and digital management for a matrix of urban communities played an important role in China. Wuhan put four categories of personnel (confirmed cases, suspected cases, febrile patients who might be carriers, and close contacts) under classified management in designated facilities. The measure was subsequently carried out nationwide. The digital management for a matrix of urban communities has become the most important component of residents’ quarantine. Many communities across China rallied the family doctor, neighborhood committees, police, and other forces to establish workgroups. The workgroup was fully responsible for community prevention and control of epidemics [17]. Each community kept only one entrance and exit point open, and checkpoints were set up for community staff to perform the identification and temperature tests of each resident entering and leaving the community. Community management is the first line of defense in the battle against infectious diseases. This is an important means to control the spread of infectious diseases from the source.

Singapore has continuously upgraded its border control measures to curb the import of overseas cases. With the implementation of multi-pronged surveillance strategy, patients can be found as quickly and accurately as possible.

Through the classified management of “four categories of personnel” and digital management for a matrix of urban communities, China has taken good control of the community as a defense line. China earnestly promoted “Leave no one unattended”, and strictly controlled the source of infection. The core of prevention and control policies in both countries is the early detection and isolation of patients. Countries can slow the spread of the virus only by actively detecting cases, identifying patients as soon as possible, and quarantining them.

Our study compared key outbreak response measures in China and Singapore and confirmed which policies are effective and can be replicated as successful experiences in other countries. We also have some limitations that deserve mentioning. COVID-19 case data were obtained from the web-page of The National Health Commission of the People’s Republic of China and the Ministry of Health Singapore. Despite some assurance of official data quality, underreporting or untimely reporting may still occur in the early stages of an outbreak, and asymptomatic carriers may be missed by symptom-based surveillance.

## Conclusions

The WHO still designates the current COVID-19 epidemic as a “Public Health Emergency of International Concern”. Countries around the world still need sustained and long-term investment to deal with the epidemic. China and Singapore’s strategies suggest that early detection of patients, timely isolation, and treatment are important means to control the spread of the virus. In addition, it is necessary to increase medical capabilities and ensure adequate medical resources to combat COVID-19. Countries should choose appropriate response strategies with health equity in mind to ultimately control effectively the spread of COVID-19 worldwide.

## Data Availability

No additional data are available.
